# Household Food Waste Patterns Across Groups: A Clustering Analysis Based on Theory of Planned Behavior Constructs and Shopping Characteristics

**DOI:** 10.3390/foods14223883

**Published:** 2025-11-13

**Authors:** Xuerui Yang, Catherine G. Campbell, Cody Gusto, Kathleen D. Kelsey, Helen Haase, Kai Robertson, Nevin Cohen, Gregory A. Kiker, Ziynet Boz

**Affiliations:** 1 Department of Family, Youth and Community Sciences, Institute of Food and Agricultural Sciences, University of Florida, Gainesville, FL 32611, USA; yang.x1@ufl.edu (X.Y.); cgusto@ufl.edu (C.G.); 2 Department of Agricultural Education & Communication, University of Florida, Gainesville, FL 32611, USA; 3 Department of Horticultural Sciences, University of Florida, Gainesville, FL 32611, USA; kathleen.kelsey@ufl.edu; 4 Department of Agricultural and Biological Engineering, University of Florida, Gainesville, FL 32611, USA; haasehelen@ufl.edu (H.H.); gkiker@ufl.edu (G.A.K.); ziynetboz@ufl.edu (Z.B.); 5 Department of Computer Science, University of Applied Sciences Hamburg, 20099 Hamburg, Germany; 6 Independent Researcher, Washington, DC 20007, USA; robertson.kai@gmail.com; 7 CUNY Graduate School of Public Health and Health Policy, New York, NY 10027, USA; nevin.cohen@sph.cuny.edu; 8 CUNY Urban Food Policy Institute, New York, NY 10027, USA

**Keywords:** food waste, k-medoid clustering, shopping habits, survey, demographics and socioeconomics

## Abstract

Theory of planned behavior (TPB) constructs and shopping routines are strong predictors of food waste behavior, while socio-demographic factors show mixed and weaker associations. We analyzed survey data from a nationally representative sample of 1066 U.S. households, measuring self-reported food waste frequency across meals, food types, and disposal methods. We applied k-medoid clustering on 19 TPB constructs and 25 shopping characteristics to identify three distinct consumer segments. “Structured Planners” (Cluster 1) showed the most deliberate shopping habits and strongest engagement in food waste reduction. “Flexible Planners” (Cluster 2) shared similar waste outcomes but approached shopping with greater spontaneity, while “Younger Wasters” (Cluster 3) were younger, lower-income, and less educated, with casual shopping habits, lower ratings of TPB constructs, and the highest food waste frequency overall. These distinct behavioral profiles enable policymakers to directly identify and target specific demographic segments for tailored food waste interventions. Particularly, “Younger Wasters” reported a significantly higher food waste frequency at 6.7 times per week, while “Structured Planners” and “Flexible Planners” were statistically similar at approximately 4.6 and 4.4 times per week. Dinner is the meal resulting in the most food waste across all groups, and “Younger Wasters” reported the highest frequency of waste in protein, oil, and grain. Post-clustering ANOVA analysis tested the predictive power of TPB, shopping characteristics, and cluster membership on food waste frequency. Results show that “Younger Wasters”, along with variables like attitude, store shopping frequency, and shopping behavior, are significantly positively associated with food waste frequency. This study demonstrates the potential of clustering analysis in exploring food waste determinants and suggests using clustered indices as proxies for respondents’ overall traits.

## 1. Introduction

Food waste poses a substantial concern due to its direct and indirect economic and environmental impacts. In the United States (U.S.), 66 million tons of food loss and waste are generated in the retail and consumer sectors. Sixty percent (39 million tons) of wasted food goes to landfills [1]). Food waste creates a direct loss of food value estimated at $243 billion and $728 per capita annually in the U.S. [2]. Driven by the urgent need for more research on food waste, the field has seen rapid growth in studies from social, agricultural, and environmental sciences. Principato et al. reviewed 233 such papers published between 2002 and 2018, with over half appearing in just the last two years [3].

Building on this growing research base, recent studies have focused on understanding the behavioral and social mechanisms driving consumer-level food waste behavior (FWB). The factors statistically associated with FWB include the theory of planned behavior (TPB) constructs and shopping characteristics associated with the waste that results from consumer food management process, which is often referred to as the “squander sequence.”. Although studies and interpretation of the above factors on FWB are well-documented, factors related to socio-economic status and demographics (which we collectively refer to as “socio-demographic factors”) may also provide additional influence beyond the effects of TPB constructs and shopping behavior [4]. However, the role of socio-demographic factors on shaping FWB remains mixed in the literature: the magnitudes and directions of the relationships with FWB vary between studies, resulting in little practical wisdom for reducing food waste in the U.S. [5,6,7,8].

The mixed literature regarding socio-demographic factors poses a challenge: while some studies find lower-income households waste more food, others report opposite findings (richer people waste more). This inconsistency impedes the development of targeted interventions, as it is unclear which population groups that policymakers should prioritize. The conflicting findings may arise from the assumption that socio-demographic factors directly determine food waste, when, in fact, their effect may interconnect with shopping and psychological factors. This study has two primary objectives: (1) to identify distinct consumer segments based solely on TPB and shopping characteristics using clustering, and (2) to examine whether these behaviorally defined segments exhibit different FWB across meal types, food categories, and disposal methods, without pre-imposing socio-demographic variables or waste behavior categories in the clustering settings. To address these objectives, we conducted a survey of a nationally representative sample of U.S. households in September 2024. This survey collected information about their psychological factors regarding food waste using TPB, as well as grocery shopping-related factors, socio-demographic traits, and frequency of food waste.

## 2. Food Waste Behavior: Behavioral Theory and Methodology

While several behavioral theories have been applied to consumer food waste contexts, TPB has served as a foundational framework in understanding food waste behaviors at the household level. Many studies successfully applied the TPB in food waste and found that the core TPB constructs—attitudes (one’s positive or negative evaluation of a given behavior), belief (practical benefits of food waste is positively with food waste), subjective norms (the social pressure one perceives they are receiving from others to perform or not perform a behavior), and perceived behavioral control (one’s perception of how easy or difficult it is to perform a behavior given internal or external constraints)—were all significantly associated with study participants’ intent to separate food waste [9,10,11].

Food waste occurs at four stages, which collectively can be referred to as the “squander sequence”: pre-acquisition, acquisition, consumption, and disposition (i.e., disposal) [12]. While the squander sequence (and comparable descriptive models of consumer food waste) provides empirically grounded justifications for focusing on each stage along the sequence, prior research placed emphasis on the significance of the pre-acquisition and acquisition stages. This emphasis is due to the fact that shopping characteristics, including planned or spontaneous choice of stores, frequency of visits, and purchasing or acquisition of food items (among other actions), are considered crucial determinants of household food waste [13,14].

As for socio-demographics, evidence shows that younger people tend to waste more food [15,16,17]. However, the relationship between socio-demographics and food waste remains controversial. Some studies have found that lower income is associated with less reported food waste [7], while others observed the opposite [6], or no significant relationships [5]. Demographic and socioeconomic variables have frequently been regarded as background factors in food waste studies due to these discrepancies and the indirect role that demographic factors play within psychological frameworks [18].

Traditional regressions that estimate the marginal effects of socio-demographic variables often rely on strong model specifications, such as assumptions of linearity, independence, and absence of multicollinearity. When these assumptions are violated or, when the sample is not well-suited, model misspecification can occur, leading to biased estimates or the attribution of significance to inappropriate variables. Consequently, prior research has shown that socio-demographic effects on FWB tend to be inconsistent or indirect.

Rather than continuing to examine the marginal effects of socio-demographic variables on FWB, this study employed clustering from which to view the findings. Clustering is a person-centered, descriptive method that groups individuals according to shared motivational patterns [19,20]. This differs from variable-centered approaches, which treat individuals as alike except for the specific values they take on model variables.

By clustering well-established direct predictors (TPB constructs and shopping characteristics) rather than socio-demographics or waste outcomes, we test whether the behaviorally homogeneous groups naturally align with socio-demographic profiles. If they do, this would explain why imposing socio-demographics as independent predictors produces inconsistent results. The cluster membership then serves as a composite indicator capturing the joint influence of socio-demographic backgrounds, which we evaluate alongside TPB and shopping characteristics in predicting food waste through ANOVA.

In this case, we group respondents based on well-established predictors of food waste (namely, TPB constructs and shopping characteristics) and then investigate how these clusters differ in terms of socio-demographics and FWB. Together with the initial TPB and shopping characteristics, we also evaluate the cluster membership’s statistical contribution to food waste prediction using ANOVA.

Lastly, we measured FWB based on the three dimensions: frequency by meal types, frequency by food types, and disposal methods. Each dimension captures a unique household waste behavioral stage. It is important to note that food waste varies according to the context in which food is prepared and consumed. Prior studies show that waste of perishable foods is less related to shopping characteristics [18,21], while non-perishable foods such as grains are more related to shopping. High-bread-wasting households buy more bread per shopping trip in Norway and Germany [22,23]. Similarly, examining waste generated at different meals complements the analysis of waste by type of food, as mealtime routines can drive the waste of specific foods (e.g., bread at breakfast or produce at dinner), and reveal more context-specific FWB. Disposal methods represent the final stage of waste management decisions, reflecting households’ varying levels of waste awareness and management practices across consumer segments.

Our approach allows us to explore the determinants of FWB through basic descriptive comparisons: summarizing the clustering inputs TPB and shopping characteristics within each cluster, and comparing them against the outcome variable of FWB, along with external variables of interest like socio-demographics. Combining with clustering and ANOVA, we offer an alternative to previous attempts for modeling of psychological, food related routines, and socio-demographics in FWB such as regression [10], principal component analysis [24], k-means clustering [25], and confirmatory factor analysis [7]. This alternative may also help reveal how a person’s internal traits such as socio-demographics are naturally connected to their TPB responses and shopping characteristics.

Therefore, this study clusters respondents including core TPB constructs and shopping characteristics from pre-acquisition and acquisition stages (planning, store frequency, store-related, and during-shopping factors) to better understand FWB patterns. Socio-demographics are external factors used for descriptive statistical comparison with the TPB and shopping characteristics in the post-clustering analysis.

## 3. Methods

### 3.1. Sample Recruitment

We acquired a nationally representative sample (NRS) of U.S. households through the Qualtrics survey platform to strategically recruit participants based on key socio-demographic variables such as age, income, and education. This research was approved by the University of Florida Institutional Review Board (IRB# ET00019640) on 17 July 2023. Recruitment proceeded over a 7-day period, with the final sample consisting of 1115 enrolled respondents. Prior to completing the survey, respondents were informed of their rights as research participants and presented with an informed consent form that clearly outlined the purpose of the study, confidentiality and data security protocols, and the voluntary nature of their participation. This form was built into the opening section of the survey, and all respondents were required to indicate that they had read and agreed to the terms before proceeding.

### 3.2. Food Waste Measures

FWB was measured using three categories of self-reported frequency measures, each representing a distinct dimension of household food waste (see Table 1).

The first category is food waste by meal types (4 variables), capturing how frequently households discard food during breakfast, lunch, dinner, and snack times during a typical week.

The second category is food waste disposal methods (5 variables), measuring how frequently households discard food in specific ways, including feeding pets, backyard composting, community composting, garbage disposal, and throwing away in trash.

The third category is food waste by food types (9 variables), calculating how frequently households discard specific food categories, including egg protein, meat-based protein, plant-based protein, dairy, fruits, vegetables, grains, fats/oils, and inedible parts (e.g., eggshells, bones).

The survey operationalized the definition of food waste at the beginning of the survey as follows: “When we refer to “food waste” in this survey we mean any time the following are thrown in the trash, composted or otherwise discarded”, and provided specific examples to each food type (e.g., “Vegetables (e.g., leafy greens; carrots; squash)”, “Plant-based protein (e.g., nuts; seeds; tofu)”).

For the “inedible parts” food type, participants were instructed at the beginning of the survey that food waste includes “inedible foods and drinks parts of foods typically considered inedible (such as eggshells or coffee grounds) and spoiled food items.” This instruction, combined with the examples provided (e.g., eggshells, bones), guided respondents to classify items like peelings as inedible parts based on their typical household practices and cultural norms. Complete examples of food types are available in Table S1b. Hence, the examples in the survey likely provided sufficient guidance for consistent classification in typical scenarios, though we acknowledge that some edge cases (e.g., vegetables cooked in oils) may have been classified differently by different respondents.

The FWB variables are measured in 5-point Likert frequency: (e.g., “Less than once a week” to “Every day of the week”). For analysis purposes, these ordinal responses were converted to numerical values representing days per week (see Table S1a for scale anchors).

Furthermore, we tested the internal consistency of the three categories of FWB measures using Cronbach’s alpha. Table S6 presents good to excellent internal consistency: food waste by meal (α = 0.84, 95% CI [0.82, 0.85]), food waste by type (α = 0.93, 95% CI [0.92, 0.94]), and food waste disposal (α = 0.75, 95% CI [0.73, 0.78]). Meanwhile, our approach to using self-reported frequency measures in food waste research for large-scale surveys is consistent with established practices in the field (see Table S6). We discuss limitations of self-reported frequency measures in Section 5.1.

### 3.3. Clustering Analysis

We conducted the clustering analysis using a subset of 44 variables from the data. The subset included 19 TPB core factors and 25 factors related to shopping characteristics, such as shopping planning, store frequency, reasons for store choice, and in-store habits (See Table 1 and Table S1a in Supplementary Materials). We excluded socio-demographic variables to test whether clusters defined by psychological and shopping characteristics would naturally align with demographic patterns without imposing this relationship. We also excluded FWB variables to avoid circular reasoning that would arise from clustering on food waste measures and then examining food waste differences between clusters. By using only 44 TPB and shopping variables as clustering inputs, we could objectively examine whether these segments exhibit different food waste patterns as outcomes, while cluster membership serves as a composite indicator for underlying socio-demographics variation.

The data used in the clustering consisted of a mix of ordinal and numerical variables. Most TPB and shopping characteristics were treated as ordinal, as they were measured using 5-point or 8-point Likert scales. To appropriately handle the ordinal-based data types, we employed Gower’s distance—a metric shown to be more robust than Euclidean distance in this case. Technical details are shown in Supplementary Material.

After calculating the Gower’s distance triangular matrix for the complete sample, we applied the Partitioning Around Medoids (PAM) algorithm to minimize the average distance between respondents and their nearest medoid representative [26]. Compared to k-means or k-modes clustering, PAM can directly utilize the Gower’s distance matrix we calculated.

**Figure 1:**
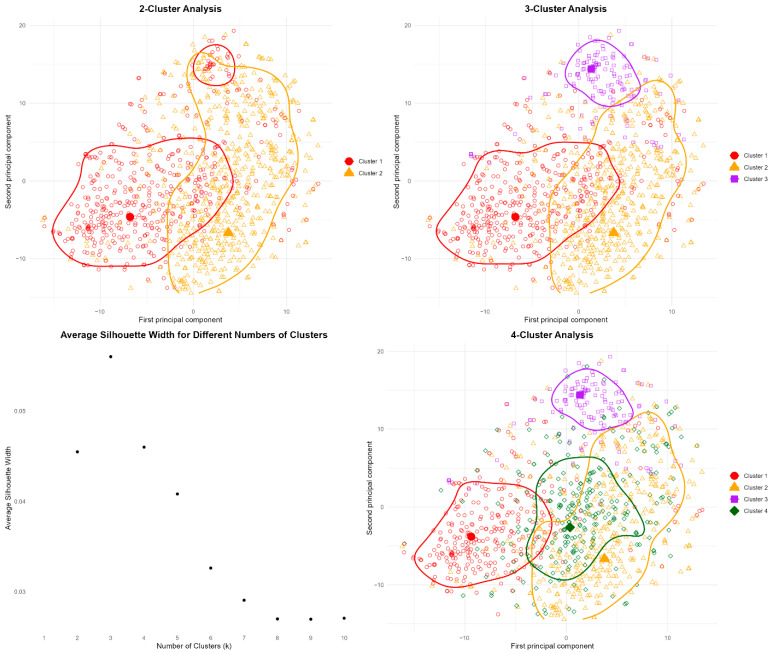
Clustering analysis of a 3-cluster solution. Note: The figure shows 44 TPB and shopping characteristics as a 2D principal component. The large, solid points are the medoids selected by the PAM algorithm. The hollow points represent individual respondents, and the ellipses delineate cluster boundaries.

Based on Silhouette score and inspection of the contour-line visualizations, we determined that a 3-cluster solution provided optimal performance for our PAM clustering approach [27]. The analysis shows the “evolutionary” pattern of an increasing number of medoids in partitioning the respondents. In the 2-cluster solution, we identified a group of respondents located far from either medoid, suggesting they form a distinct third group. The 4-cluster solution performed poorly, with the fourth cluster’s medoid positioned too close to Cluster 2’s solution, failing to create meaningful separation. The silhouette analysis confirmed 3 clusters as optimal, achieving the highest silhouette score (see Figure 1 and Supplementary Material Figure S3 for a detailed 3-cluster visualization).

Specifically, these contour lines were constructed by applying a 2D kernel density estimation on the t-SNE projected points and then overlaying the resulting density levels on the scatterplot. For clarity, we showed only the outermost contour line for each cluster. Although it does not directly show the exact percentage of points included, the outermost line roughly captures the broader shape of the cluster. All clustering procedures were implemented using functions from the “cluster” and “Rtsne” packages in R [25,28,29].

### 3.4. Between-Cluster Comparison

**Figure 2:**
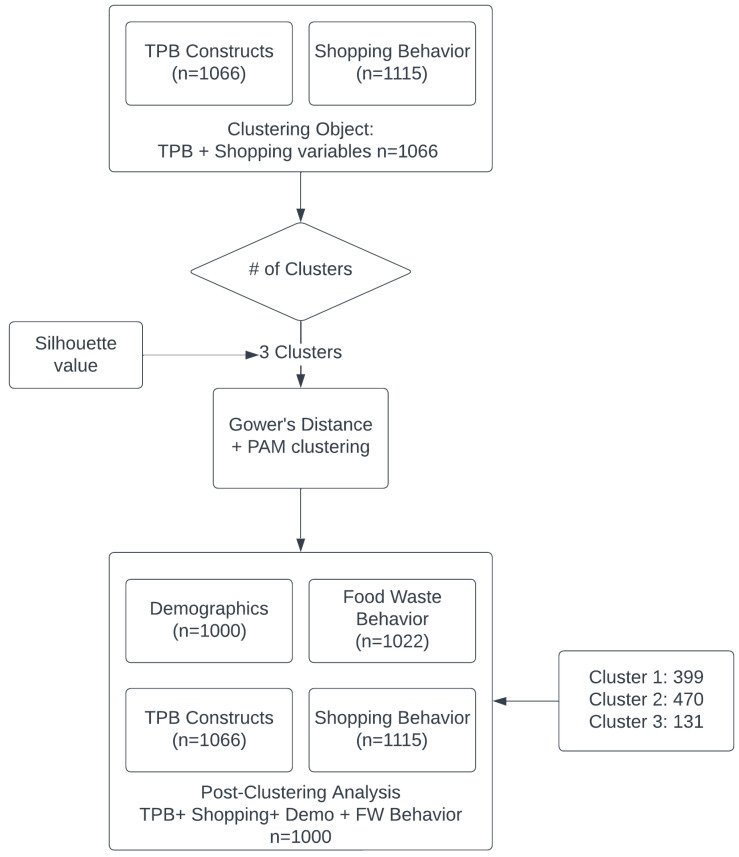
National Representative Sample Inclusion Flow for Clustering and Post-Clustering Analyses. Note: # indicates number of observations/households.

The initial NRS of 1115 respondents contained some missing or questionable responses (For example, some respondents answered “yes” to having children in the household but then reported “0” in the follow-up question on the number of children) which we recoded as missing and excluded from relevant analyses. As a result, the TPB constructs had 1066 complete responses (4.4% missing), while the shopping characteristics had no missing values. Since TPB and shopping characteristics were used as clustering inputs, we used the 1066 complete cases for clustering to ensure the most stable and reliable clusters and to retain maximum statistical power (see Figure 2).

Once participants were grouped into three clusters (Clusters 1, 2, and 3), we conducted the post-clustering analysis by comparing 1: TPB constructs, 2: shopping characteristics, 3: demographics, and 4: FWBs across clusters. Since FWBs and demographics displayed more missing values (8.3–10.3% missing), we limited the analysis to a subset of approximately 1000 respondents with complete data across all relevant domains.

For the TPB constructs and shopping characteristics, we compared the means and standard deviations of each factor across the clusters. We also examined how socio-demographic factors and FWB varied across clusters. Like the FWB variables, TPB constructs were measured using 5-scale ordinal scales (e.g., “Strongly disagree” to “Strongly agree”) Table 1 summarizes all key measures used in this study.

### 3.5. ANOVA of TPB, Shopping on Food Waste

Lastly, we conducted a series of ANOVA models to examine the determinants of FWB. Specifically, we assessed the effects of core TPB constructs, pre-shopping characteristics and during-shopping characteristics. In addition, we included a “Cluster” factor derived from the earlier clustering. “Cluster” captures individuals’ within-cluster differences in FWB. Since TPB constructs and shopping are controlled, this factor mainly serves a composite indicator for the distinct socio-demographic backgrounds.

$$\begin{array}{c} FW\;Frequency=\mu +Cluster\;2+Cluster\;3+ATT_{j}+{BB}_{k}+IN_{l}+PC_{m}+\\ SP_{n}+SF_{p}+SR_{q}+SB{\;Plan}_{r}+SB\;{Imp}_{t}+\epsilon \end{array}$$

where ATT reflects average attitude toward food waste, BB captures behavioral beliefs, IN represents injunctive norms, and PC measures perceived behavioral control, all following the TPB framework; SP refers to pre-shopping behaviors, SF is shopping frequency for specific stores; SR captures store-related shopping motivations;

Planned shopping behaviors (SB Plan) refer to behaviors associated with deliberate planning and reduced food waste, consisting of “Buy only items on your shopping list” (SB1) and “Check date labels on perishable items” (SB6).

Impulsive shopping behaviors (SB Imp) linked to overbuying and increased waste risk. It includes four constructs, such that “Buy food in larger quantities than desired due to the way food is packaged” (SB2), “Buy in bulk for lowest unit price” (SB3), “Purchase more due to sales or BOGO deals” (SB4), and “Purchase something unplanned” (SB5). These categorizations align with prior research showing that list adherence and date checking reduce waste, while impulse and bulk buying increase waste risk [6].

Each of the predictors corresponds to a rescaled 3-point factor (except for shopping frequency: SF) to simplify the illustration of regression output, as the original 5-point Likert scale would be too detailed for display purposes. These factors were calculated as the average across items within the same category of factors (see Table S1a for item-level detail and Table S4 for calculation details).

## 4. Results

### 4.1. Cluster Formation and Sample Distribution

The optimal 3-cluster solution partitioned 1066 observations into Cluster 1 (n = 424), Cluster 2 (n = 499), and Cluster 3 (n = 143). The complete post-clustering analysis included 1000 observations, with missing values distributed evenly across clusters, resulting in Cluster 1 (n = 399), Cluster 2 (n = 470), and Cluster 3 (n = 131). The missing data was removed in roughly proportional amounts across all three clusters, indicating no systematic bias in data completeness by cluster type.

### 4.2. Diversity of TPB and Shopping

Since the medoid of clusters is an actual respondent in the NRS and serves as the typical profile of each cluster, we identified the medoid of each cluster and described the characteristics of the TPB constructs and shopping characteristics, respectively. Based on both the TPB constructs and shopping characteristics, can be summarized as follows: Cluster 1—proactive waste reducers with organized shopping habits; Cluster 2—proactive waste reducers with flexible shopping habits; and Cluster 3—low waste engagement with casual shopping habits. Hence we labelled Clusters 1, 2, 3 as “Structured Planners”, “Flexible Planners”, and “Younger Wasters” (see “Diversity of Demographics”), respectively.

First, the medoids of Cluster 1 and Cluster 2 share similar proactive attitudes across all TPB-related questions. In contrast, the Cluster 3 medoid gave mostly neutral answers, reflecting a more indifferent or disengaged attitude across the TPB constructs (see Table 2).

Second, the main differences between Cluster 1 and Cluster 2 medoids appear in their shopping characteristics. Cluster 1 shows characteristics of taking a more deliberate and organized approach to food shopping. They place high importance on planning and during-shopping activities, such as checking what’s already in the pantry, making a shopping list, or checking date labels on perishable items. Cluster 1 respondents also highly value store experience factors like customer service, affordability, product quality and variety, and cleanliness. In contrast, Cluster 2 takes a more relaxed approach to shopping, planning less consistently and having more casual expectations for stores. Though Cluster 2 acknowledges these factors matter, they simply do not prioritize them as much as Cluster 1 does.

Lastly, Cluster 3’s shopping habits show some distinct patterns. In both planning and during-shopping characteristics, Cluster 3 is similar to Cluster 2, as both groups tend to plan less and pay less attention to in-store details. But unlike Cluster 2, they care less about store features and shop differently. This may be because Cluster 3 has lower incomes. They still think store quality matters somewhat, but not as much as the other groups. Basically, while Cluster 3 might want the same things as Cluster 2, their shopping decisions are more driven by what they can afford and what’s practical. We discussed the distribution of socio-demographics across the clusters later.

Although medoid offers a useful snapshot of a “typical” consumer within each cluster, not all individuals behave exactly like the medoid. For instance, even though the Cluster 2 medoid rated “Type of people who shop at the store” as “Not at all important,” many others in the same cluster felt differently. To show this variation, we included the full distributions of responses for all TPB and shopping characteristics in Figures S1 and S2. Looking specifically at the factor “Type of people who shop at the store” (SP7, Figure S2), more than half of Cluster 2 respondents selected “Not important”. It is a higher proportion than in Clusters 1 and 3, highlighting shared tendencies, even if not perfectly reflected by the medoid. Finally, we included the summary statistics of these variables for the pre-clustering NRS in Tables S2 and S3, for easier comparison with the post-clustering pattern in each cluster.

### 4.3. Diversity of Demographics

Table 3 reports a summary of demographic distribution by cluster. We conducted statistical tests of independence to assess differences across clusters. Results revealed no significant demographic differences between Cluster 1 and Cluster 2 (all *p* > 0.05). On the contrary, Cluster 3 differed significantly from Clusters 1 + 2 combined across seven demographic variables: income (*p* < 0.001), age (*p* < 0.001), education (*p* < 0.001), employment status (*p* = 0.009), SNAP food assistance participation (*p* = 0.002), census region (*p* = 0.048), and gender (*p* = 0.011).

The racial and ethnic makeup of the respondents is comparable across all three clusters with no significant differences (*p* = 0.706): approximately 61% are non-Hispanic white, followed by Hispanic and African American people. In each cluster, a significant percentage of respondents (72.7% to 80.5%) also stated that they did not have any children living in the home, with no significant differences across clusters (*p* = 0.176).

Beyond these similarities, Cluster 3 stands out demographically, representing a younger, lower-income group that is less engaged in food waste reduction. Specifically, Clusters 1 and 2 show an even income distribution, with roughly one-third of respondents falling into each bracket: “under $50,000”, “$50,000–$109,999”, and “over $110,000”. In contrast, Cluster 3 skews lower income, with 59.6% earning less than $50,000 and only 11.5% earning above $110,000.

Distributions of ages also vary. Compared to 32.1% in Cluster 3, the percentage of younger respondents (under 40) is 17.5% in Cluster 1 and 13.0% in Cluster 2. At the same time, 54.2% of Cluster 1, 51.1% of Cluster 2, and 38.9% of Cluster 3 are composed of elderly persons (60+).

Alongside the income and age differences, Cluster 3 shows a different educational profile, as shown by the higher percentage with “High school or less” education and lower percentage with “Graduate or professional degree.” Moreover, Cluster 3 has double the unemployed percentage of the other two clusters. Given the age distribution, Clusters 1 and 2 include more retired respondents. Interestingly, “employed” percentages are similar across all three groups.

The regional distribution of the NRS sample was evaluated by classifying respondents by U.S. Census areas. Across all clusters, the Midwest and Northeast were consistently represented: roughly 21% for the Midwest and 17–19% for the Northeast as opposed to Cluster 3, which had more respondents from the South (33%) and fewer from the West (26%), Clusters 1 and 2 had more respondents from the West (35–37%) and fewer from the South (23–25%).

### 4.4. Diversity of Food Waste Behavior

There were three types of questions for the respondents associated with FWB: 1. food waste frequency by meal types, 2. food waste disposal frequency, and 3. food waste frequency by food types.

#### 4.4.1. Total Food Waste and Meal Types

Table 4 lists the mean frequency of each FWB and the test results of differences in means across the clusters. We first compared the mean frequency between Cluster 1 and Cluster 2, then combined Clusters 1 and 2, and finally compared this combined group with Cluster 3.

We examined the estimated food waste by meal types. On average, respondents reported the highest waste frequency during dinner: 1.69 times per week in Cluster 1, 1.76 in Cluster 2, and 2.19 in Cluster 3. The overall weekly food waste frequency was 4.59 for Cluster 1, 4.42 for Cluster 2, and 6.66 for Cluster 3. Statistical tests confirmed that there were no significant differences in food waste frequency between Cluster 1 and Cluster 2 at the 5% significance level, for any of the meal types or for total weekly waste. However, Cluster 3 reported significantly more food waste than Clusters 1 and 2 in almost every meal category and in total waste, except for breakfast. If this reported frequency accurately reflects actual food waste, Cluster 3, the younger, low-income group with the least waste engagement and casual shopping habits, wasted more food than Clusters 1 and 2. The latter group included older, more affluent individuals who were more proactive in reducing food waste, with mixed shopping habits: some were organized, while others were more casual.

#### 4.4.2. Food Waste Disposal

Among disposal behaviors, the three clusters demonstrated notable differences in how they manage food waste once generated. Cluster 3 showed a higher frequency of garbage disposal use than the other clusters in every category. However, “community compost” was the only category that was statistically higher for Cluster 3 compared to the other two clusters. As for the “throw in the trash” option, the three clusters showed no significant difference, and it remained the most commonly used method overall, averaging about 2.2 times per week.

#### 4.4.3. Food Waste by Food Types

Cluster 3 reported more frequent food waste compared to Clusters 1 and 2. Moreover, Clusters 1 and 2 demonstrated statistically similar behaviors in both waste frequency and disposal practices. This pattern extends to food waste by type. Cluster 3 reported a consistently higher frequency across all food categories (see Table 4, “Average” row in the last block), with an average of 1.54 times per week, compared to 1.26 and 1.10 times for Clusters 1 and 2. These averages by food type are also comparable to waste frequency by meal, which was 1.15, 1.11, and 1.67 times per week for Clusters 1, 2, and 3, respectively (see “Average” in the first block).

Looking more closely at specific food types, Cluster 3 reported statistically higher frequencies of wasting proteins such as “Egg,” “Meat,” “Plant,” and “Dairy,” as well as “Grains and Oil or Fat”, compared to the other two clusters. In contrast, there were no significant differences among the clusters in the categories of “Fruit,” “Vegetables,” and “Inedible” food waste.

### 4.5. ANOVAs: Validity of Clusters and the Role of Shopping Characteristics

ANOVA results highlight two important points. First, even when controlling TPB and shopping characteristics, the cluster variable still significantly explains differences in food waste frequency. This supports the meaningfulness of the clustering results. Second, shopping characteristics, especially store shopping frequency and behavior during shopping, were statistically significant. One TPB construct, attitude, also showed a significant relationship with food waste frequency, as shown in the left panel of Table 5.

The regression estimates in Table 5’s right panel report that Cluster 3 individuals reported significantly higher food waste levels than those in Clusters 1 and 2. Clusters 1 and 2 were not statistically different from each other. Moreover, at least one level within several variables, including attitude, belief, store shopping frequency, and both planned and impulsive shopping characteristics, was significantly associated with food waste frequency. Since the goal of the ANOVA is to validate the effect of the cluster factor and shopping characteristics in explaining food waste, along with TPBs, discussing the estimated relationships between factors and food waste frequency within the scope of this study.

## 5. Discussion

### 5.1. Clustering Results: TPB Validation and Socio-Demographic Patterns

Our findings support several theories in the literature. First, they validate the TPB’s utility in explaining consumers’ perceptions of food waste. Our clustering results confirmed that respondents with more proactive attitudes, beliefs, social norms, and perceived behavioral control reported lower frequencies of food waste than those in the more passive, less engaged cluster. This result is consistent with previous research showing that the TPB’s core constructs are useful in explaining FWB, particularly elements that are driven primarily by psychological factors such as self-awareness or individual intention [9,12].

Beyond TPB validation, our clustering approach revealed distinct socio-demographic patterns. We explored whether clustering based on behavioral and psychological factors would naturally align with specific socio-demographic profiles. The younger, less-educated, lower-income, and predominantly male group (Cluster 3) shows lower TPB agreement toward food waste reduction, more unplanned and casual shopping habits, and the highest reported food waste frequency. The ANOVA results showed that cluster membership remained significantly associated with higher food waste, even after controlling for TPB and shopping characteristics. Specifically, Cluster 3 membership demonstrated a significant association with increased food waste behavior.

Through the clustering process, we described the critical traits of socio-demographics across the three clusters. When combined with their TPB responses and shopping characteristics, a consistent pattern emerged: individuals who were younger and had lower incomes tended to express less concern toward food waste. This suggests that clustering based on food waste core motivations may help uncover socio-demographic patterns that might otherwise show mixed or weak associations with food waste in traditional regressions [6,7,8]. Clustering takes a person-centered view by revealing how demographic traits interconnect with broader motivational and behavioral patterns [30]. Rather than treating age, income, or education as isolated predictors, this approach highlights how such traits intersect and combine within clusters that reflect common social and economic contexts. These demographic patterns also varied geographically. The West, has the highest median income among U.S. regions, was more represented in Clusters 1 and 2. Meanwhile, the South–home to the lowest regional incomes—was more prominent in Cluster 3 [31]. This geographic distribution further reinforces the income-related patterns observed in our clusters.

Our clustering results aligned older and wealthier individuals with Clusters 1 and 2, groups who were more motivated to reduce food waste. This finding is particularly noteworthy given that prior research has found limited statistical evidence linking income or age to self-reported food waste once TPB-related factors are controlled for in regression models [10,32,33].

### 5.2. Shopping Characteristics and Food Waste Relationships

The relationship between shopping behaviors and food waste is more complex than that of socio-demographics. Although the clustering results suggest that Clusters 1 and 2 report similar levels of food waste despite having distinct shopping patterns (organized versus casual), the ANOVA results provide a clearer picture of which specific shopping characteristics drive food waste.

Specifically, more frequent store shopping and impulsive shopping behaviors, such as responding to buy-one-get-one-free promotions, are significantly associated with higher self-reported waste frequency. These relationships are well-established in previous studies [32,34,35]. In contrast, planned shopping behaviors, such as checking labels and buying only items on a shopping list, are negatively associated with waste frequency, which is also supported by Ahmed et al. [36].

Thus, the ANOVA results aligned with previous studies on the “squander sequence” or “food waste journey” [3,12]. These patterns are well-supported by marketing and psychological theories, such as the planning fallacy, present value bias, and optimism bias [37].

In summary, although clustering revealed broad behavioral patterns across consumer segments, the ANOVA results provide more granular evidence about the influence of shopping behavior on food waste frequency. ANOVA analysis showed that individual shopping behaviors, such as frequency of store visits and responsiveness to promotions, are significant predictors of waste even after controlling for cluster membership. The clustering approach captured overall shopping styles (organized versus casual), which did not significantly differentiate waste levels between Clusters 1 and 2. These results imply that both strategies provide complementing insights: ANOVA reveals the precise behavioral levers most strongly linked to food waste, while clustering identifies customer segments with unique motivational characteristics.

### 5.3. Waste Disposal Behaviors

Cluster 3’s disposal patterns reveal an unexpected paradox that warrants further examination. On the one hand, Cluster 3 has the lowest food waste reduction engagement and the highest reported food waste frequency among all clusters. On the other hand, they also report more frequent use of environmentally friendly disposal methods that might offset some of the negative consequences of their FWB. One possible explanation is the “licensing effect”. This occurs when waste-mitigating behaviors such as composting or feeding food scraps to pets make consumers feel more comfortable discarding food, as these positive actions reduce their guilt about wastefulness [12,38].

However, a closer examination reveals a more complex issue. While composting is generally regarded as environmentally friendly and helps maintain soil fertility [39,40], it is primarily intended for fruit and vegetable-based food waste. Cluster 3’s high frequency of protein and oil waste contradicts their reported use of backyard and community composting, as these facilities are not suited for protein and oil-based items like meat, dairy, and fats [41]. Additionally, protein-based foods are not only unsuitable for composting but are also expensive to treat and contribute significantly to air, soil, and water pollution due to their high biological and chemical oxygen demands [42]. Kamal et al. also maintained the challenges in converting protein food waste into fertilizer or animal feed [43].

This mismatch between waste types and disposal methods suggests the licensing effect may not fully explain Cluster 3’s behavior. It is possible that these respondents were not aware that their reported disposal practices may not be appropriate for the types of food they are wasting. Future research that directly links food waste types with their actual disposal methods would help clarify this behavior and help determine whether consumers understand which disposal methods are appropriate for different food types, such as directing vegetables and fruits to composting facilities and oils and proteins being diverted to pets, livestock, or other appropriate channels.

## 6. Conclusions and Implications

This study leverages a person-centered clustering approach to provide a clearer understanding of household food waste patterns than traditional variable-centered methods. We clustered TPB constructs and shopping characteristics which are established direct predictors of food waste. Then, we found that socio-demographic distinctions naturally reflected underlying behavioral patterns. This finding explains the inconsistent demographic effects reported in prior literature: socio-demographic factors affecting food waste through interconnected behavioral pathways, instead of independent predictors in model specifications.

The clustering approach successfully identified three behaviorally homogeneous segments that align with distinct demographic profiles and waste patterns, with cluster membership effectively functioning as a unified proxy for the complex interplay of socio-demographic characteristics. This approach helps researchers and policymakers to identify behaviorally grounded segments that can guide more precisely targeted interventions, serving as an alternative to regression model specifications. Instead of treating age, income, or education as independent variables with assumed linear effects, our approach recognizes that these characteristics act together within lived social contexts to shape food waste behaviors.

### 6.1. Theoretical Implications

In previous food waste studies, clustering and other segmentation methods were used as a terminal approach to investigate the relationships among food waste measures and other determinants [44]. In contrast, we regarded clustering as a middle step to group households based on TPB constructs and shopping characteristics, not food waste measures. Instead, the identity of clustered households is assigned by socio-demographics after clustering. Using this strategy, we conceptually bypassed the modelling specification between socio-demographics and food waste measures, by using cluster variables as membership, to investigate the relationship between three categories of food waste measures.

Our results extend food waste theory in two ways. First, the natural alignment between socio-demographics and TPB and shopping patterns validates our methodological approach and suggests a new research paradigm. This alignment emerges without demographic input in the clustering. Future food waste studies may first identify behavioral groups using segmentation methods, then examine their demographic composition.

Second, FWB should not be assumed to be uniform across food types. The selective increase in proteins, dairy, grains, and fats/oils in Cluster 3, but not fruits or vegetables, challenges aggregate waste measures. Future research may account for this variation rather than relying solely on aggregate food waste measures.

### 6.2. Practical and Policy Implications

The heterogeneity in food waste patterns across food types has important practical implications. The protein-specific waste pattern in Cluster 3 (younger, lower-income households) means interventions should be tailored to target specific food categories rather than adopting “one-size-fits-all” approaches. For example, campaigns targeting Cluster 3 might focus on proper storage and portion control for proteins, while campaigns for other segments might emphasize different food categories.

More broadly, this study demonstrates that clustering, as a person-centered method, has strong potential in classifying the targeted population for food waste reduction.

The findings showed that Cluster 3 wasted the most because of a series of socio-demographic factors—not due to any single factor such as low education, unemployment, or low income, but rather through the intercorrelated impacts that jointly link Cluster 3 to such wasteful behavior. While the knowledge–attitude–behavior framework explains partial motivations behind behavior (such as knowledge being associated with education and income), it does not directly pinpoint which population needs campaigns or policy interventions focusing on practical skills such as meal planning, portioning, and safe food storage. Hence, this study not only benefits researchers by allowing them to “control” for a group of socio-demographic variables in food waste studies but also provides a straightforward landscape for policymakers to target populations with a set of “typical” profiles.

### 6.3. Limitations and Future Research

Two major limitations stem from the sample structure. First, FWB in this study was self-reported. Second, the unit of measurement was frequency, rather than volume or weight. As a result, the ANOVA estimates primarily reflect what respondents consciously recognize and are willing to discard. However, prior studies have emphasized that a substantial portion of food waste occurs unintentionally. Respondents may underreport food waste if it is unconscious or goes unnoticed, which would reduce any correlation between food waste and upstream behaviors like shopping habits. Moreover, a significant share of actual waste may fall outside respondents’ awareness or reporting. Therefore, frequency, as a self-reported measure, is less precise than volume or weight [10,12], Still, this approach represents a practical compromise for a large, nationally representative survey of over 1000 respondents, and it remains consistent with much of the leading literature in this area, where frequency or proportion scales have been widely applied, shown in Table S6.

We encourage future researchers to distinguish between conscious and unconscious food waste when interpreting the behavioral mechanisms that drive it. In addition, we advocated future studies to conduct food waste measures across food types. One important caveat is ensuring that participants interpret the measures consistently. Future studies should provide clearer instructions for classifying food types to reduce ambiguity in edge cases (e.g., whether vegetables cooked in oil should be counted as vegetable waste or inedible waste).

## Figures and Tables

**Table 1:** Summary of Key Measures Used in Analysis.

Variable Domains	Role in Analysis	Number of Variables	Scale	Example Variables
Clustering Analysis (PAM clustering)				
TPB Constructs	Clustering input	19	5-point Likert	Attitudes, Beliefs, Norms, Control
Shopping characteristics	Clustering input	25	5-point or 8-point Likert	Planning, Store frequency, Shopping reasons
Post-clustering Analysis (Between-Cluster Comparison)				
TPB Constructs	Compared across clusters	19	5-point Likert	(same as above)
shopping characteristics	Compared across clusters	25	5-point or 8-point Likert	(same as above)
Socio- demographics	Compared across clusters	9	factor	Income, Age, Education, Employment
Food Waste Behavior				
Food Waste by Meal	Outcome compared across clusters	4	Numerical	Breakfast, Lunch, Dinner, Snack
Food Waste by Type	Outcome compared across clusters	9	Numerical	Proteins, Dairy, Grains, Vegetables
Food Waste Disposal	Outcome compared across clusters	5	Numerical	Pets, Compost, Disposal, Trash
Post-clustering Analysis (ANOVA)				
Cluster Membership	predictor	2	Binary	Cluster 2, Cluster 3 (vs. Cluster 1)
TPB Constructs	predictor (rescaled)	19	3-point Likert	(aggregated from original)
shopping characteristics	predictor (rescaled)	25	3-point or 4-point Likert	(aggregated from original)
Food Waste by Meal	Dependent variable (total)	1	Numerical	Total weekly food waste frequency

Note: The full description of each variable characteristic is listed in Table S1a in Supplementary Materials.

**Table 2:** Cluster Medoids Across TPB Constructs and Shopping Characteristics.

Category	Cluster 1	Cluster 2	Cluster 3
TPB Constructs			
Attitudes (ATT1-ATT6)	Positive	Positive	Neutral
Beliefs (BB1–BB8)	Agree	Agree	Neither agree nor disagree
Norms (IN1–IN3)	Agree	Agree	Neither agree nor disagree
Perceived Control (PC1–PC2)	Agree	Agree	Neither agree nor disagree
Shopping Characteristics			
Shopping Planning (SP)	Always plan and list	Sometimes plan and list	Sometimes plan and list
Store Frequency (SF)	Weekly groceries, never discount store	Weekly groceries, rare discount store	Monthly groceries, frequent discount store
Shopping Reasons (SR)			
Product quality, cleanliness, deals	Extremely important	Very important	Moderately important
Convenience & location	Very important	Very important	Moderately important
Customer service	Extremely important	Very important	Very important
Store atmosphere	Very important	Very important	Moderately important
People type in stores	Moderately important	Not at all important	Moderately important
Technology & pickup	Not important	Not important	Moderately important
Shopping Behavior (SB)	Always follow lists and checks labels	Sometimes follow lists and check labels	Sometimes follow lists and check labels

Note: The full description of each variable characteristic is listed in Tables S1a and S5 in Supplementary Materials.

**Table 3:** Demographic Characteristics of Three Clusters.

Variable	Cluster 1 (n = 399)	Cluster 2 (n = 470)	Cluster 3 (n = 131)
Income level ***	**Proportion (%)**		
Under $50,000	36.3	33.8	58.8
$50,000–$109,999	33.3	37.9	29.8
More than $110,000	30.3	28.3	11.5
Age range (years) ***			
Under 40	17.5	13.0	32.1
40–59	30.6	33.2	29.0
60+	51.9	53.8	38.9
Education level ***			
High school or less	17.8	16.0	35.9
Some college/technical/vocational	24.3	20.4	22.1
Associate degree	11.8	12.1	12.2
Bachelor’s degree	26.8	28.7	23.7
Graduate or professional degree	19.3	22.8	6.1
Children living in the home			
Yes	24.3	19.5	27.3
No	75.7	80.5	72.7
Ethnicity and Race			
Black or African American	10.2	10.4	13.8
Asian	6.5	5.5	6.2
Hispanic	18.2	18.3	16.2
Non-Hispanic White	61.0	61.8	61.5
Other (e.g., American Indian)	4.0	4.0	2.3
Employment status **			
Employed	46.1	40.1	39.4
Unemployed	11.0	12.7	22.7
Retired	39.6	41.6	31.8
Student	1.0	1.1	3.0
Other	2.3	4.5	3.0
Received food assistance in last 12 months (SNAP) **			
Yes	26.1	22.3	38.2
No	72.7	76.4	60.3
Don’t know	1.3	1.3	1.5
Census region *			
Midwest	21.1	21.7	21.4
Northeast	17.5	17.7	19.1
South	25.6	23.0	33.6
West	35.8	37.7	26.0
Gender			
Female	74.4	72.8	64.9
Male	25.6	27.2	34.4
Non-binary	0.0	0.0	0.8

Note: Chi-square tests (or Fisher’s exact test when expected cell counts < 5) were conducted to compare demographic distributions across clusters. Asterisks indicate significant differences between Cluster 3 and Clusters 1 + 2 combined: * *p* < 0.05, ** *p* < 0.01, *** *p* < 0.001. Between Cluster 1 and Cluster 2: No significant demographic differences were found (all *p* > 0.05). Between Cluster 3 and Clusters 1 + 2 combined: Cluster 3 differed significantly from Clusters 1 + 2 combined for income, age, education, employment, SNAP participation, census region, and gender. Variables without asterisks (Children living in the home, Ethnicity and Race) showed no significant differences.

**Table 4:** Mean Weekly Frequency of FWB Across Three Clusters with Statistical Comparisons.

	Mean (SD)		
Variable	Cluster 1 (n = 399)	Cluster 2 (n = 470)	Cluster 3 (n = 131)
Breakfast	1.07 (2.15)	0.94 (1.89)	1.29 (1.94)
Lunch	1.03 (2.04)	0.93 (1.76)	1.48 ** (1.93)
Dinner	1.69 (2.16)	1.76 (2.05)	2.19 * (2.27)
Snack	0.79 (1.90)	0.79 (1.75)	1.69 *** (2.27)
Average	1.15 (1.88)	1.11 (1.65)	1.67 ** (1.80)
Total	4.59 (7.50)	4.42 (6.59)	6.66 ** (7.19)
Feed to Pets	1.41 (2.42)	1.12 (1.95)	1.57 (2.29)
Backyard compost	0.89 (1.97)	0.92 (1.96)	1.16 (1.92)
Community compost	0.71(1.86)	0.59(1.58)	1.05 * (1.91)
Garbage disposal	1.50 (2.23)	1.38 (2.04)	1.74 (2.22)
Throw away in trash	2.23 (2.37)	2.17 (2.15)	2.28 (2.21)
Egg protein (e.g., egg yolk)	0.78 (1.88)	0.60 (1.48)	1.19 ** (1.97)
Meat-based protein (e.g., fish; chicken)	1.08 (1.91)	0.99 (1.68)	1.46 * (1.97)
Plant-based protein (e.g., nuts; seeds)	0.70 (1.74)	0.63 (1.47)	1.22 ** (1.88)
Dairy (e.g., cheese; butter)	1.07 (2.08)	0.80 * (1.62)	1.37 * (2.08)
Fruits (e.g., strawberries)	1.39 (2.07)	1.19 (1.69)	1.45 (2.07)
Vegetables (e.g., leafy greens)	1.36 (2.00)	1.30 (1.68)	1.68 (2.01)
Grains (e.g., rice; bread)	1.13 (2.08)	0.84 * (1.62)	1.42 * (1.95)
Fats and/or oils (e.g., lard; cooking oils)	1.21 (2.06)	1.03 (1.77)	1.74 ** (2.19)
Inedible parts (e.g., eggshells; bones)	2.59 (2.57)	2.49 (2.31)	2.32 (2.32)
Average	1.26 (1.70)	1.10 (1.31)	1.54 (1.68)

Note: Values represent self-reported frequency in days per week. * *p* < 0.05, ** *p* < 0.01, *** *p* < 0.001. Asterisks in Cluster 2 column indicate significant differences between Clusters 1 and 2. Asterisks in Cluster 3 column indicate significant differences between Cluster 3 and Clusters 1 + 2 combined. “Inedible parts” refers to any portions of food that participants discarded, such as by throwing in the trash, feeding to animals, using a garbage disposal, composting, or other means. These include parts typically considered inedible (e.g., eggshells, coffee grounds) as well as spoiled food items.

**Table 5:** ANOVA with TPB Constructs and Shopping Characteristics on Self-Reported Food Waste Frequency.

TPB Constructs	Estimate	S.E.	Shopping	Estimate	S.E.
Cluster			Shopping Planning		
Cluster 1 (Ref)			Less than Half (Ref)		
Cluster 2	0.38	0.47	Half the time	0.47	0.68
Cluster 3	2.06*	0.87	More than Half	0.67	0.65
Attitude			Shopping Frequency		
Negative (Ref)			Less Often (Ref)		
Neutral	−0.28	1.12	Monthly to Weekly	1.39 **	0.45
Positive	−2.17 *	1.01	Several Times a Week	5.27 ***	0.85
			Daily	13.09 ***	1.55
Belief			Shopping Reasons		
Disagree (Ref)			Unimportant (Ref)		
Neutral	−2.52 *	1.23	Neutral	−1.01	0.9
Agree	−1.84	1.22	Important	−0.43	0.91
Injunctive Norms			Planned Shopping Behavior		
Disagree (Ref)			Less than Half (Ref)		
Neutral	−0.54	0.81	Half the time	−1.83	
Agree	−0.1	0.75	More than Half	−1.56 **	
Perceived Behavior Controls			Impulsive Shopping Behavior		
Disagree (Ref)			Less than Half (Ref)		
Neutral	−0.25	1.07	Half the time	0.75	
Agree	−0.92	0.94	More than Half	3.12 ***	
R-squared	0.1933				
Observation	1000				

Note: Outcomes represent self-reported total food waste frequency in days per week. * *p* < 0.05, ** *p* < 0.01, *** *p* < 0.001. Rescaling and details for TPB Constructs and Shopping Variables are listed in Table S4 in Supplementary Materials.

## Data Availability

All data used in this study will be made available upon reasonable request. The lead author has full access to all the data reported in the manuscript and takes responsibility for the integrity and accuracy of the analyses.

## Supplementary Materials

The following supporting information can be downloaded at https://www.mdpi.com/article/10.3390/foods14223883/s1, Table S1a: List of Variable Descriptions and Non-Missing Observations (TPB and Shopping); Table S1b: List of Variable Descriptions and Non-Missing Observations (Food Waste); Table S2: The Summary Statistics for TPB Constructs in the National Representative Sample (N = 1066); Table S3: The Summary Statistics for Pre-Shopping, Store Choice, and During Shopping Behavior in the National Representative Sample (N=1,066); Table S4: Rescaling of 5-Point Likert Scale Items to 3-Point Scale for TPB Constructs and Shopping Variables; Table S5: Details of Cluster Medoids Across Key Demographic, TPB Constructs, and Shopping Behavior; Table S6: Examples of Self-Reported Food Waste Measures in Prior Studies; Figure S1: TPB Constructs by Three Clusters; Figure S2: Shopping and Store Behavior Profiles by Three Clusters; Figure S3: Respondents Segmentations based on TPB Constructs and Shopping Variables (Detailed View).
