# Supplementary material for: Household Food Waste Patterns Across Groups: A Clustering Analysis Based on Theory of Planned Behavior Constructs and Shopping Characteristics
Source: Foods. 2025 Nov 13;14(22):3883. doi: 10.3390/foods14223883 (PMC12650926; doi:10.3390/foods14223883)
Supplement: Supplementary file 1 [file foods-14-03883-s001.zip › foods-3924444-supplementary.pdf]

Technical Details A: Gower's Distance and PAM

Gower's distance between respondents i and j at the k-th ordinal variable (e.g., $x_{i,k}$ and $x_{j,k}$) was calculated as follows:

$$d_{i,j,k} = \frac{|x_{i,k} - x_{j,k}|}{x_{k} - x_{k}}$$

The total distance (dissimilarity) between respondents i and j is a weighted sum of all the 45 variables in the subset:

$$d_{i,j} = d(i,j) = \frac{\sum_{k=1}^{45} w_k \delta_{i,j,k} \times d_{i,j,k}}{\sum_{k=1}^{45} w_k \delta_{i,j,k}}$$

where w_k is the weight of the k-th variable, and all variables are equally weighted at default. $\delta_{i,j,k}$ is a 0/1 binary variable attached to the weight w_k, representing the comparability of the value of the k-th variable between observations i and j. So, $\delta_{i,j,k} = 0$ only if either or both observations are missing the k-th variable.

PAM randomly selects a predetermined number of observations as the initial medoids and assigns all other observations to one of these initial medoids based on the "closest" distance. The algorithm then calculated the total costs (measured in distance) for all observations under the current medoid configuration. In the following rounds, PAM systematically evaluated selecting other observations as new medoids and recalculated the total costs for each potential replacement. If a new medoid reduced the total cost compared to the current configuration, PAM will swap out the existing medoid with this new one. This process continues iteratively until no additional swaps further reduced the total cost.

Technical Details B:

These ordinal variables were recoded from ranked categories to numerical values, following the scheme presented in the first section of Table S1.

For example, participants were asked: *"How frequently do you have food waste at the following times during a typical week?"* with options ranging from *"Less than once a week"* to *"Every day of the week."*

To analyze these responses, we converted them into discrete numerical values as follows:

$$Food\ Waste\ per\ Week = \begin{cases} 0 \ \ if \text{ "Less than once a week "} \\ 1.5\ if \text{ "1 − 2 days a week"} \\ 3.5\ if \text{ 3-4 days a week} \\ 5.5\ if \text{ 5-6 days a week} \\ 7\ if \text{ "Every day of the week"} \end{cases}$$

We applied this coding to food waste reported at four meal occasions and summed them to estimate the total frequency of food waste per week.

$$FW_{Total} = FW_{Breakfast} + FW_{Lunch} + FW_{Dinner} + FW_{Snack}$$

Since the NRS did not ask about the quantity or weight of food wasted at each meal, this summation assumes equal weight for each meal type, regardless of the amount or type of food discarded.

Table S1a. List of Variable Descriptions and Non-Missing Observations (TPB and Shopping)

Variables	Description	Factor Levels
***Attitude: Reducing Food Waste Attitudes…* (1-5 Scale)**		
ATT1	Overall bad (1) – Overall good (5)	Strongly disagree (1)
ATT2	Harmful (1) – Beneficial (5)	Disagree (2)
ATT3	Useless (1) – Useful (5)	Neither agree nor
ATT4	Unimportant (1) – Important (5)	disagree (3)
ATT5	Not a priority (1) – A high priority (5)	Agree (4)
ATT6	Bad for communities (1) – Good for communities (5)	Strongly agree (5)
***Belief: Reducing Household Food Waste Beliefs…* (1-5 Scale)**		
BB1	… conserve energy.	Strongly disagree (1)
BB2	… conserve water.	Disagree (2)
BB3	… reduce food insecurity.	Neither agree nor
BB4	… decrease landfill use.	disagree (3)
BB5	… decrease greenhouse gas emissions.	Agree (4)
BB6	…Help mitigate climate change	Strongly agree (5)

BB7	… make a meaningful difference environmentally.	
BB8	… Save money	
***Injunctive Norms: Household Food Waste Injunctive Norms* (1-5 Scale)**		
IN1	People who are important to me believe I should reduce the amount of food I discard.	Strongly disagree (1)<br>Disagree (2)<br>Neither agree nor disagree (3)<br>Agree (4)<br>Strongly agree (5)
IN2	Most people important to me support my efforts to reduce the amount of food I discard.	
IN3	The media I consume frequently highlights the importance of food waste.	
***Perceived Behavioral Control: Household Food Waste Perceived Controls* (1-5 Scale)**		
PC1	I am capable of discarding less food than I currently do.	Strongly disagree (1)<br>Disagree (2)<br>Neither agree nor disagree (3)<br>Agree (4)<br>Strongly agree (5)
PC2	Whether I reduce my food waste is completely up to me.	
Variables	Description	**Factor Levels**
***Shopping Planning: Before food shopping, how often do you or members of your household …* (1-5 Scale)**		
SP2	Check to see what is in your fridge/freezer and pantry	Never (1)<br>Sometimes (2)<br>About half time (3)<br>Most of the time (4)<br>Always (5)
SP3	Plan your meals	
SP4	Make a shopping list	
SP5	Estimate how much of each item you need to buy	
***Store Frequency: How frequently do you typically purchase … from …* (1-8 Scale)**		
SP6	Superstore/"Big Box" store (e.g., Walmart; Costco)	Never (0)<br>Once a year (1)

SP7	Supermarket/Grocery store (e.g., Publix; Kroger; Albertsons)	A few times a year (2)<br>Once a month (3)<br>Once a week (4)<br>2-3 times a week (5)<br>4 or more times a week (6)<br>Daily (7)
SP8	Discount store/Convenience store (e.g., Dollar General)	
***Shopping Reasons: How important are the following factors or considerations in influencing where you purchase food?* (1-5 Scale)**		
SR1	Distance from home	Not at all important (1)<br>Slightly important (2)<br>Moderately important (3)<br>Very important (4)<br>Extremely important (5)
SR2	Convenient location	
SR3	Product affordability	
SR4	Product diversity	
SR5	Product quality	
SR6	The atmosphere of the location	
SR7	The type of people who shop at the location	
SR8	Online ordering for curbside pickup	
SR10	The overall cleanliness of the location	
SR11	Customer service	
SR12	Product deals	
SR13	Hours of operation	
***Shopping Behavior: During shopping, how often do you or members of your household do the following...* (1-5 Scale)**		
SB1	Buy only items on your shopping list	Never (1)<br>Sometimes (2)<br>About half time (3)<br>Most of the time (4)<br>Always (5)
SB2	Buy food in larger quantities than desired due to the way food is packaged	
SB3	Buy food in large quantities to get the lowest possible unit price	

SB4	Purchase more of an item than you need because it is on sale or is "buy one, get one free" (BOGO)	
SB5	Purchase something unplanned because it looks good at the time	
SB6	Check date labels (e.g., sell-by or use-by dates) on perishable items	
SB7	How many different stores do you visit during an average shopping trip?	Numerical

Table S1b. List of Variable Descriptions and Non-Missing Observations (Food Waste)

Variables	Description	Factor Levels
***Food Waste by meal: How frequently do you have food waste at the following times during a typical week* (1-5 Scale)**		
BreakfastEstimate	*Estimated Food Waste by* Breakfast	Less than once a week (1)<br>1-2 days a week (2)<br>3-4 days a week (3)<br>5-6 days a week (4)<br>Every day of the week (5)
LunchEstimate	*Estimated Food Waste by* Lunch	
DinnerEstimate	*Estimated Food Waste by* Dinner	
SnackEsimtate	*Estimated Food Waste by* Snack	
**Food waste disposal: *How frequently do you discard food in the following ways during an average week?* (1-5 Scale)**		
FW5	Feed to Pets	Less than once a week (1)<br>1-2 days a week (2)<br>3-4 days a week (3)<br>5-6 days a week (4)<br>Every day of the week (5)
FW6	Backyard compost	
FW7	Community compost	
FW8	Garbage disposal	
FW9	Throw away in trash	
**Food waste Type: *How frequently do you discard the following food types at home during an average week?* (1-5 Scale)**		
EggEstimate	Egg protein (e.g., egg yolk; egg whites)	Less than once a week (1)<br>1-2 days a week (2)<br>3-4 days a week (3)<br>5-6 days a week (4)<br>Every day of the week (5)
MeatEstimate	Meat-based protein (e.g, fish; chicken)	
PlantEstimate	Plant-based protein (e.g., nuts; seeds; tofu)	
DairyEstimate	Dairy (e.g., cheese; butter)	
FruitEstimate	Fruits (e.g., strawberries; oranges)	
VegEstimate	Vegetables (e.g., leafy greens; carrots; squash)	
GrainsEstimate	Grains (e.g., rice; bread)	

FatsEstimate	Fats and/or oils (e.g., lard; cooking oils)
InedEstimate	Inedible parts (e.g., eggshells; meat bones)

Table S2. The Summary Statistics for TPB Constructs in the National Representative Sample (N=1,066)

Variables	M	SD
***Reducing Food Waste Attitudes…* (1-5 Scale [i])**		
Overall bad – Overall good	4.40	1.10
Harmful – Beneficial	4.39	1.05
Useless – Useful	4.36	1.08
Unimportant – Important	4.44	0.97
Not a priority – A high priority	4.18	1.05
Bad for communities – Good for communities	4.34	1.04
Overall Attitude scale	4.35	0.86
***Reducing Household Food Waste Beliefs…* (1-5 Scale [i])**		
… conserve energy.	3.38	0.85
… conserve water.	3.46	0.79
… reduce food insecurity.	3.48)	0.75
… decrease landfill use.	3.70	0.62
… decrease greenhouse gas emissions.	3.42	0.81
…Help mitigate climate change	3.29	0.90
… make a meaningful difference environmentally.	3.44	0.84
… Save money	3.82	0.50
Overall Belief scale	3.50	0.55
***Household Food Waste Injunctive Norms* (1-5 Scale [i])**		
People who are important to me believe I should reduce the amount of food I discard.	3.53	0.75
Most people important to me support my efforts to reduce the amount of food I discard.	3.02	1.02

The media I consume frequently highlights the importance of food waste.	3.49	0.75
***Household Food Waste Perceived Controls* (1-5 Scale [i])**		
I am capable of discarding less food than I currently do.	3.41	0.86
Whether I reduce my food waste is completely up to me.	3.74	0.58

[i] 1=strongly disagree, 2=disagree, 3=neither agree nor disagree, 4=agree, 5=strongly agree

Table S3. The Summary Statistics for Pre-Shopping, Store Choice, and During Shopping Behavior in the National Representative Sample (N=1,066)

Variables	M	SD
***Before food shopping, how often do you do the following…* (1-5 Scale [i])**		
Check to see what is in your fridge/freezer and pantry	3.82	1.15
Plan your meals	3.29	1.19
Make a shopping list	3.87	1.28
Estimate how much of each item you need to buy	3.69	1.22
***How frequently do you typically purchase and/or acquire your food from the following sources?* (1-8 Scale [ii])**		
Superstore/"Big Box" store	4.72	2.38
Supermarket/Grocery store	5.93	2.29
Discount store/Convenience store	3.55	2.43
***How important are the following factors or considerations in influencing where you purchase food* (1-5 Scale [iii])**		
Distance from home	3.37	1.12
Convenient location	3.57	1.03
Product affordability	3.95	0.96
Product diversity	3.48	1.10
Product quality	4.21	0.85
The atmosphere of the location	3.34	1.05
The type of people who shop at the location	2.61	1.29
Online ordering for curbside pickup	2.19	1.38
The overall cleanliness of the location	3.91	0.97
Customer service	3.74	0.98
Product deals	3.83	0.98
Hours of operation	3.50	1.06
***During food shopping, how often do you do the following?* (1-5 Scale [i])**		
Buy only items on your shopping list	3.09	1.23

Buy food in larger quantities than desired due to the way food is packaged	2.46	1.02
Buy food in large quantities to get the lowest possible unit price	2.64	1.15
Purchase more of an item than you need because it is on sale or is "buy one, get one free" (BOGO)	2.81	1.14
Purchase something unplanned because it looks good at the time	2.81	1.08
Check date labels (e.g., sell-by or use-by dates) on perishable items	3.74	1.30

[i] 1=never, 2=sometimes, 3=about half the time, 4=most of the time, 5=always

[ii] 1=Never, 2=Once a year, 3=A few times a year, 4=Once a month, 5=Once a week, 6=2-3 days a week, 7=4-6 days a week, 8=Daily

[iii] 1=Not at all important, 2=Slightly important, 3=Moderately important, 4=Very important, 5=Extremely important

Table S4. Rescaling of 5-Point Likert Scale Items to 3-Point Scale for TPB Constructs and Shopping Variables

5-Scale to 3-Scale	ATT1	"Bad" (1) = "Overall Bad" (1),<br>"Bad" (1) = "Somewhat Bad" (2),<br>"Neutral" (2) = "Neither Good nor Bad" (3),<br>"Good" (3) = "Somewhat Good" (4),<br>"Good" (3) = "Overall Good" (5)
	ATT2	"Harmful" (1) = "Harmful" (1),<br>"Harmful" (1) = "Somewhat Harmful"(2),<br>"Neutral" (2) = "Neither Beneficial nor Harmful" (3),<br>"Beneficial" (3) = "Somewhat Beneficial" (4),<br>"Beneficial" (3) = "Beneficial" (5)
	ATT3	"Useless" (1) = "Useless" (1),<br>"Useless" (1) = "Somewhat Useless" (2),<br>"Neutral" (2) = "Neither Useful nor Useless" (3),<br>"Useful" (3) = "Somewhat Useful" (4),<br>"Useful" (3) = "Useful" (5)
	ATT4	"Unimportant" (1) = "Unimportant" (1),<br>"Unimportant" (1) = "Somewhat Unimportant" (2),<br>"Neutral" (2) = "Neither Important nor Unimportant" (3),<br>"Important" (3) = "Somewhat Important" (4),<br>"Important" (3) = "Important" (5)
	ATT5	"Not a Priority" (1) = "Not a Priority" (1),<br>"Not a Priority" (1) = "Somewhat not a Priority" (2),<br>"Neutral" (2) = "Neutral" (3),<br>"A Priority" (3) = "Somewhat a Priority" (4),<br>"A Priority" (3) = "A High Priority" (5)
	ATT6	"Bad for Communities" (1) = "Bad for Communities" (1),<br>"Bad for Communities" (1) = "Somewhat Bad for Communities" (2),<br>"Neutral" (2) = "Neither Good nor Bad for Communities" (3),

		"Good for Communities" (3) = "Somewhat Good for Communities" (4),<br>"Good for Communities" (3) = "Good for Communities" (5)<br>$\text{ATT Overall} = \begin{cases} 1 \text{ if } 1 < \text{Average of 6 ATTs} < 1\frac{2}{3} \\ 2 \text{ if } 1\frac{2}{3} < \text{Average of 6 ATTs} < 2\frac{1}{3} \\ 3 \text{ if } 2\,\frac{1}{3} < \text{Average of 6 ATTs} < 3 \end{cases}$
5-Scale to 3-Scale	BB1-BB8	"Disagree" (1) = "Strongly disagree" (1)<br>"Disagree" (1) = "Disagree" (2)<br>"Neutral" (2) = "Neither agree nor disagree" (3)<br>"Agree" (3) = "Agree" (4)<br>"Agree" (3) = "Strongly agree" (5)<br>$\text{BB Overall} = \begin{cases} 1 \text{ if } 1 < \text{Average of 8 BBs} < 1\frac{2}{3} \\ 2 \text{ if } 1\frac{2}{3} < \text{Average of 8 BBs} < 2\frac{1}{3} \\ 3 \text{ if } 2\,\frac{1}{3} < \text{Average of 8 BBs} < 3 \end{cases}$
5-Scale to 3-Scale	IN1-IN3	Same as BBs<br>$\text{IN Overall} = \begin{cases} 1 \text{ if } 1 < \text{Average of 3 INs} < 1\frac{2}{3} \\ 2 \text{ if } 1\frac{2}{3} < \text{Average of 3 INs} < 2\frac{1}{3} \\ 3 \text{ if } 2\,\frac{1}{3} < \text{Average of 3 INs} < 3 \end{cases}$
5-Scale to 3-Scale	PC1, PC2	Same as BBs<br>$\text{IN Overall} = \begin{cases} 1 \text{ if } 1 < \text{Average of 2 PCs} < 1\frac{2}{3} \\ 2 \text{ if } 1\frac{2}{3} < \text{Average of 2 PCs} < 2\frac{1}{3} \\ 3 \text{ if } 2\,\frac{1}{3} < \text{Average of 2 PCs} < 3 \end{cases}$
5-Scale to 3-Scale	SP2-SP5	"Less than Half" (1) = "Never" (1),<br>"Less than Half" (1) = "Sometimes" (2),<br>"Half the time" (2) = "About half the time" (3),<br>"More than Half" (3) = "Most of the time" (4),

	"More than Half" (3) = "Always" (5)<br>$$\text{SP Overall} = \begin{cases} 1 \text{ if } 1 < \text{Average of 4 SPs} < 1\frac{2}{3} \\ 2 \text{ if } 1\frac{2}{3} < \text{Average of 4 SPs} < 2\frac{1}{3} \\ 3 \text{ if } 2\frac{1}{3} < \text{Average of 4 SPs} < 3 \end{cases}$$
8-Scale to 4-Scale	SP6-SP8 "Less Often" (1) = "Never" (1),<br>"Less Often" (1) = "Once a year" (2),<br>"Less Often" (1) = "A few times a year" (3),<br>"Monthly to Weekly" (2) = "Once a month" (4),<br>"Monthly to Weekly" (2) = "Once a week" (5),<br>"Several Times a Week" (3) = "2-3 times a week" (6),<br>"Several Times a Week" (3) = "4 or more times a week" (7),<br>"Daily" (4) = "Daily" (8)<br>$$\text{SF Overall} = \begin{cases} 1 \text{ if } 1 < \text{Average of 3 SPs} < 1.75 \\ 2 \text{ if } 1.75 < \text{Average of 3 SPs} < 2.5 \\ 3 \text{ if } 2.5 < \text{Average of 3 SPs} < 3.25 \\ 4 \text{ if } 3.25 < \text{Average of 3 SPs} < 4 \end{cases}$$
5-Scale to 3-Scale	SR1-SR8, SR10-SR13 Same as ATT4<br>$$\text{SR Overall} = \begin{cases} 1 \text{ if } 1 < \text{Average of 12 SPs} < 1\frac{2}{3} \\ 2 \text{ if } 1\frac{2}{3} < \text{Average of 12 SPs} < 2\frac{1}{3} \\ 3 \text{ if } 2\frac{1}{3} < \text{Average of 12 SPs} < 3 \end{cases}$$
5-Scale to 3-Scale	SB1, SB6 Same as SP2-SP5<br>$$\text{SB Good Overall} = \begin{cases} 1 \text{ if } 1 < \text{Average of 2 SBs} < 1\frac{2}{3} \\ 2 \text{ if } 1\frac{2}{3} < \text{Average of 2 SBs} < 2\frac{1}{3} \\ 3 \text{ if } 2\frac{1}{3} < \text{Average of 2 SBs} < 3 \end{cases}$$
	SB2-SB5 Same as SP2-SP5

5-Scale to 3-Scale	$\text{SB Bad Overall} = \begin{cases} 1 \text{ if } 1 < \text{Average of 4 SBs} < 1\frac{2}{3} \\ 2 \text{ if } 1\frac{2}{3} < \text{Average of 4 SBs} < 2\frac{1}{3} \\ 3 \text{ if } 2\frac{1}{3} < \text{Average of 4 SBs} < 3 \end{cases}$

Note: Please refer to Table A.1 for variable coding names. For example, SR1 could be decoded as "distance to home" given the context of Table A.1

Table S5. Details of Cluster Medoids Across Key Demographic, TPB Constructs, and Shopping Behavior

Variable	Cluster 1 Medoid	Cluster 2 Medoid	Cluster 3 Medoid
Attitude Variables: Reducing Food Waste Attitudes…			
ATT1	Overall Good	Overall Good	Neither Good nor Bad
ATT2	Beneficial	Beneficial	Neither Beneficial nor Harmful
ATT3	Useful	Useful	Neither Useful nor Useless
ATT4	Important	Important	Neither Important nor Unimportant
ATT5	A High Priority	A High Priority	Neutral
ATT6	Good for Communities	Good for Communities	Neither Good nor Bad for Communities
Belief Variables: Reducing Household Food Waste Beliefs…			
BB1-8	Agree	Agree	Neither agree nor disagree
Injunctive Norms: Household Food Waste Injunctive Norms			
IN1-3	Agree	Agree	Neither agree nor disagree
Perceived Behavioral Control: Household Food Waste Perceived Controls			
PC1-2	Agree	Agree	Neither agree nor disagree
Shopping Planning: Before food shopping, how often do you or members of your household …			
SP2: Check left in pantry	Always	Sometimes	About half the time
SP3: Plan meals	Always	Sometimes	Sometimes

SP4-5: shopping list & Plan buying quantity	Always	Most of the time	Sometimes
Store Frequency: How frequently do you typically purchase … from …			
SP6: Superstore	Once a month	Once a month	Once a month
SP7: Grocery store	Once a week	Once a week	Once a month
SP8: Discount store	Never	A few times a year	Once a month
Shopping Reasons How important are the following factors or considerations in influencing where you purchase food?			
SR1: Distance from home	Moderately important	Very important	Slightly important
SR2: Convenient location	Very important	Very important	Moderately important
SR3-5 (Product), SR10 (clean),12 (deal),13 (operation time)	Extremely important	Very important	Moderately important
SR6: Store atmosphere	Very important	Very important	Moderately important
SR7: other people type	Moderately important	Not at all important	Moderately important
SR8: online pickup	Not at all important	Not at all important	Moderately important
SR11: Customer service	Extremely important	Very important	Very important
Shopping Behavior: During shopping, how often do you or members of your household do the following...j			
SB1: only-shopping list	Always	Sometimes	Sometimes
SB2-5	Sometimes	Sometimes	Sometimes

SB6: Check date labels	Most of the time	Sometimes	Sometimes
SB7: of Store visited	2	1	2

Note: Variable names are abbreviated (e.g., ATT1, SB1–SB9) for brevity. For full descriptions of each variable, please refer to Appendix Table A.1.

Table S6. Examples of Self-Reported Food Waste Measures in Prior Studies

Study	Food Waste Behavior Question	Response Scale / Measurement
**This study**<br>Food Waste by meal<br>(Food waste Type)	How frequently do you have food waste at the following times (discard the following food types at home) during a typical week	'Less than once a week (1)<br>1-2 days a week (2)<br>3-4 days a week (3)<br>5-6 days a week (4)<br>Every day of the week (5)<br>5-likert scale
Stefan et al., (2013). Avoiding food waste by Romanian consumers: The importance of planning and shopping routines	How much … would you say that you throw away, of what you buy and/or grow, in a regular week	'*not at all*', '*less than a tenth*', '*more than a tenth but less than a quarter*', …<br>5-likert scale
Graham-Rowe et al., (2015). Predicting household food waste reduction using an extended theory of planned behaviour	what percentage of your household's total fruit/vegetables got thrown away in the last seven days	ranged from 0% [1]–100%[11] with ten percent increments.<br>10-likert scale
Stancu, V., Haugaard, P., & Lähteenmäki, L. (2016). Determinants of consumer food waste behaviour: Two routes to food waste. Appetite, 96, 7-17.	How much … is thrown away in your household of what you buy and/or grow, in a regular week	*hardly any (1), less than a tenth (less than 10%) (2), more than a tenth but less than a quarter (between 10% and 25%) (3)…*<br>5-likert scale
Russell et al., (2017). Bringing habits and emotions into food waste behaviour.	How regularly … food is thrown away in your household	(1 = Never, 5 = Most mealtimes)<br>5-likert scale
Aydin & Yildirim (2021). Understanding food waste behavior: The role of morals, habits and knowledge.	How much … is thrown away in your household of what you buy in a regular week	(hardly any (1), less than a tenth (less than 10%) (2), more than a tenth but less than a quarter (between 10% and 25%) (3)…<br>5-likert scale

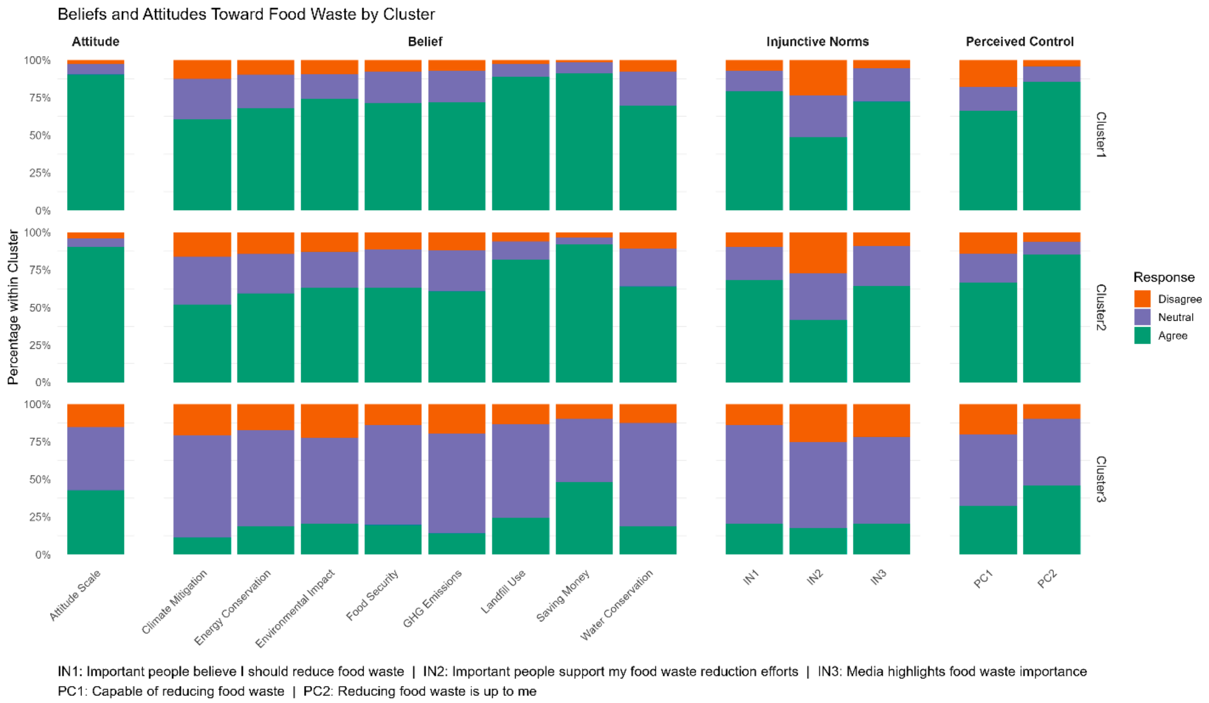

Figure S1. TPB Constructs by Three Clusters

Note: Original 5-point Likert scale was collapsed into a 3-point scale for visualization purposes. For example: “Agree (4)” and “Strongly agree (5)” were combined into “Agree”.

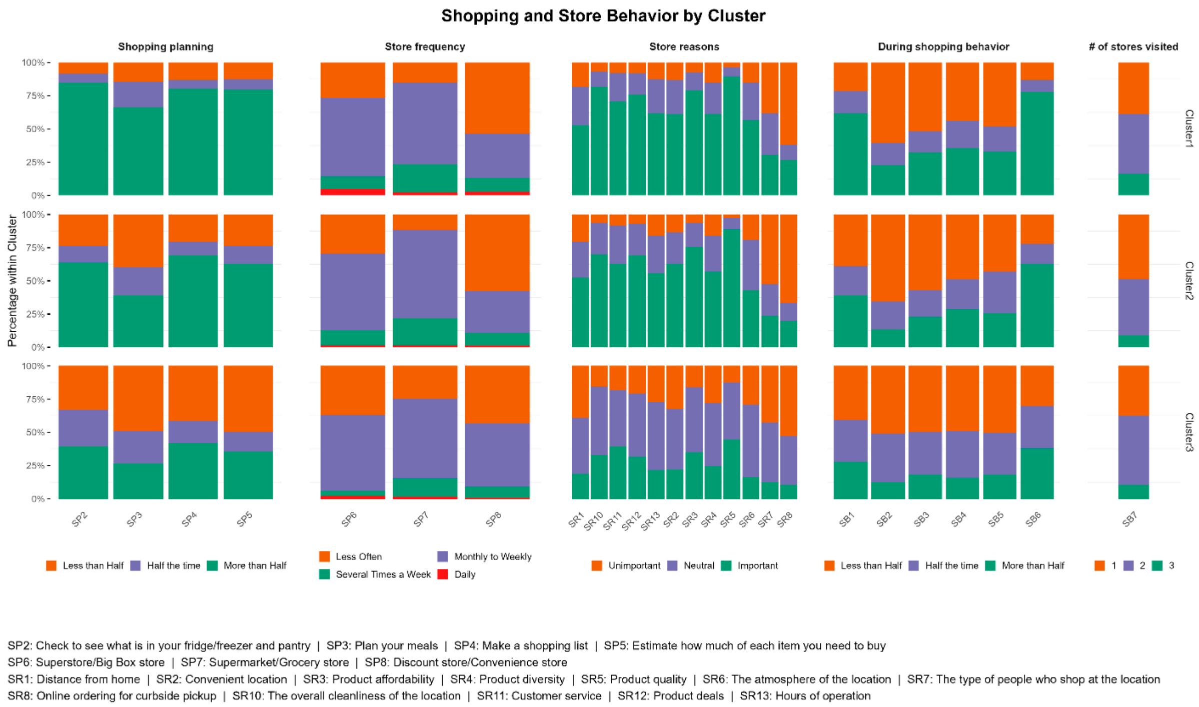

Figure S2. Shopping and Store Behavior Profiles by Three Clusters

Note: Original 5-point and 8-point Likert scales were collapsed into 3-point and 4-point scales for visualization clarity. For example, “Never (1)”, “Once a year (2)”, and “A few times a year (3)” were combined into “Less Often”.

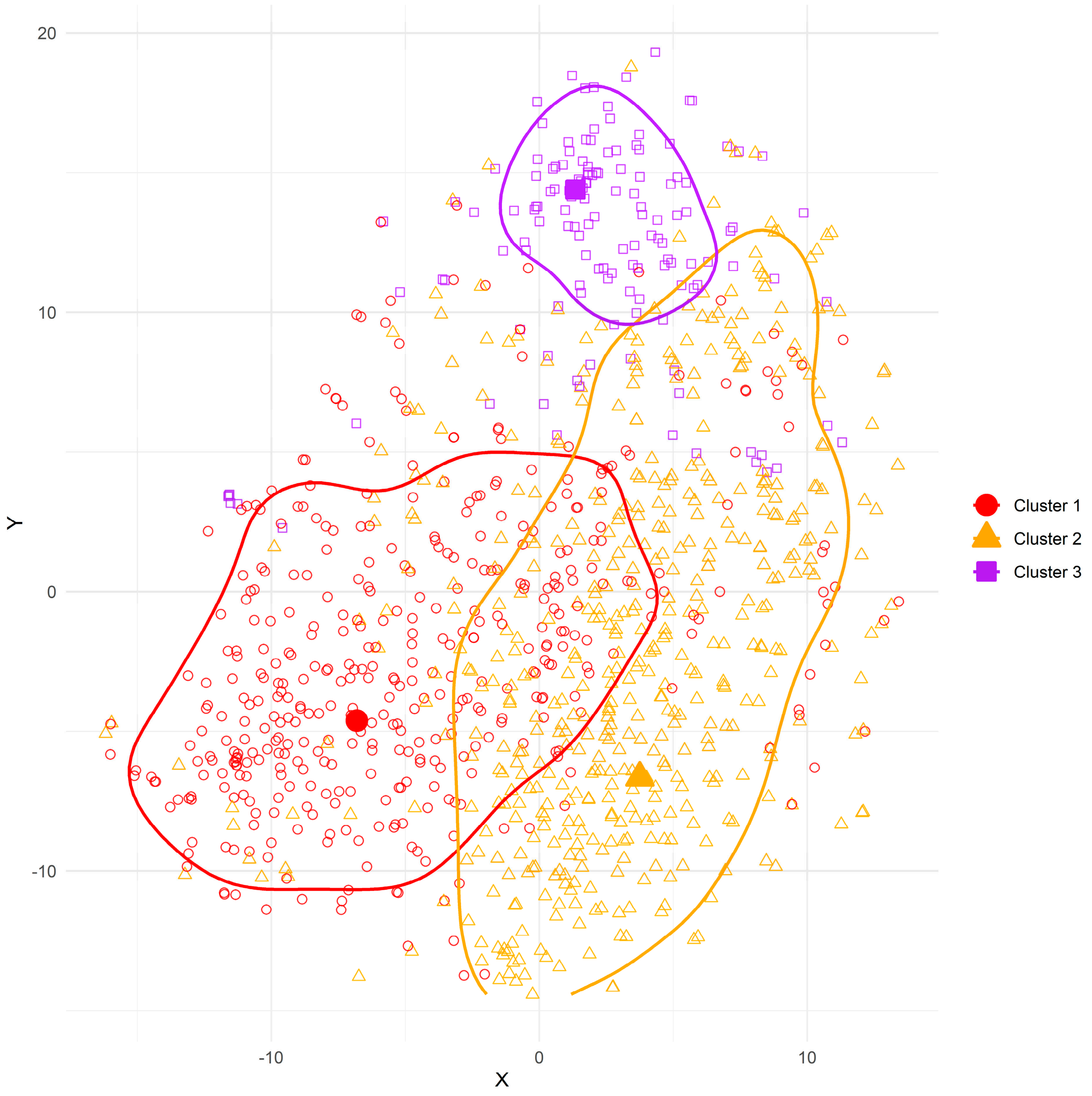

Figure S3. Respondents Segmentations based on TPB Constructs and Shopping Variables (Detailed View)
